# Interaction between categorical knowledge and episodic memory across domains

**DOI:** 10.3389/fpsyg.2014.00584

**Published:** 2014-06-11

**Authors:** Pernille Hemmer, Kimele Persaud

**Affiliations:** Department of Psychology, Rutgers UniversityPiscataway, NJ, USA

**Keywords:** episodic memory, categorical knowledge and expectations, memory tasks, categorical domains, prior knowledge, rationality

## Abstract

Categorical knowledge and episodic memory have traditionally been viewed as separate lines of inquiry. Here, we present a perspective on the interrelatedness of categorical knowledge and reconstruction from memory. We address three underlying questions: what knowledge do people bring to the task of remembering? How do people integrate that knowledge with episodic memory? Is this the optimal way for the memory system to work? In the review of five studies spanning four category domains (discrete, continuous, temporal, and linguistic), we evaluate the relative contribution and the structure of influence of categorical knowledge on long-term episodic memory. These studies suggest a robustness of peoples’ knowledge of the statistical regularities of the environment, and provide converging evidence of the quality and influence of category knowledge on reconstructive memory. Lastly, we argue that combining categorical knowledge and episodic memory is an efficient strategy of the memory system.

## INTRODUCTION

Reconstruction from memory relies not only on incomplete representations stored in memory, but also on categorical knowledge learned from past experiences. As such, many aspects of our experiences do not have to be episodically remembered, but can be extrapolated from our categorical knowledge. For example, when recalling the last time you visited a coffee shop, you might utilize not only the episodic information related to that specific visit, but also general knowledge and experiences accumulated over many coffee shop visits. You might recall that you ordered a tall coffee, not because you have detailed episodic memories, but because you have knowledge that coffee sizes are called tall, grande, and venti, and you typically order tall coffees. In this example, it is apparent that categorical knowledge can be used to facilitate recall, however, you can also imagine how this knowledge can lead to errors if, for example, the last time you visited the coffee shop you were really tired, and ordered a venti instead. Although using knowledge for that specific visit would result in error, using categorical knowledge to aid memory is on average an advantageous strategy. Filling in noisy memory with category knowledge can improve recall performance, and without it, recalling the coffee shop visit might be beyond the available episodic representation.

In this review, we proffer a perspective on the relations among categorical knowledge^1^ and episodic memory. Although the assertions of more traditional approaches in memory research, such as false memory, are that prior knowledge results in errors, the sum total of the findings presented in this review suggest that this is not the complete story. We address three underlying questions: What knowledge do people bring to the task of remembering? How do people integrate that knowledge with episodic memory when the information to be remembered is noisy and incomplete? Is this the optimal way for the memory system to work? We first address each question broadly, and then present a review of previous investigations on the interactions of episodic memory and categorical knowledge across four categorical domains encompassing discrete, continuous, temporal and linguistic categories. Each domain highlights interesting features of categorical expectations.

## WHAT DO PEOPLE BRING TO THE TASK OF REMEMBERING?

People bring a wealth of knowledge and expectations to a variety of cognitive tasks. Beginning with Sir Francis Bartlett’s (1932) seminal research, it is evident that people use knowledge of cultural and social norms, as well as cognitive expectations of the environment, to facilitate performance across tasks. Furthermore, the knowledge that people possess is well-calibrated to the statistical regularities of the natural world (e.g., Huttenlocher et al., 1991; Huttenlocher et al., 2000; Griffiths and Tenenbaum, 2006; Brady and Oliva, 2008). For example, people’s predictions about the statistics of everyday events—such as expected life spans, movie runtimes, and cake baking times – are on average quite accurate relative to the true statistics of these events (Griffiths and Tenenbaum, 2006).

An interesting property of expectations is that they can translate into domains with which people have limited experience, to further assist cognitive performance. This is consistent with the idea that statistical learning can operate at a categorical level and can be generalized to novel categories, novel exemplars, and/or categories for which people have little statistical knowledge (e.g., Brady and Oliva, 2008; Jern and Kemp, 2013). This provides a compelling argument for people’s sensitivity to the regularities of the environment, which extends across several domains of perception and cognition, including visual perception (Eckstein et al., 2004; Epstein, 2008; Todorovic, 2010), object recognition (Biederman, 1972; Palmer, 1975; Biederman et al., 1982; Hollingworth and Henderson, 1998; Torralba, 2003), object categorization (Galleguillos and Belongie, 2010), color perception (Mitterer and de Ruiter, 2008), preparing motor actions (Nissen and Bullemer, 1987), and parsing language (Trueswell, 1996).

Here, we review studies from four category domains (i.e., discrete, continuous, temporal, and linguistic) that quantify peoples’ categorical knowledge and expectations. Each task measures the signal thought to be based on the categorical representations in the mind of the observers and is consistent with categorical knowledge. The most notable finding across domains was that people expressed a surprising level of agreement, with both the expectations of others and the true stimulus distribution. In addition, each domain provided further insight into the structure of categorical expectations. In the discrete domain, salience and prototypicality were important factors for knowledge of categorical relationships. Discrete category knowledge was measured via a task where participants provided lists of all the objects associated with particular natural scene types, e.g., kitchens and offices (Hemmer and Steyvers, 2009c; Steyvers and Hemmer, 2012). Responses reflected the intuitive notion that central and salient objects in a scene have high response frequencies and that certain scene types are associated with very iconic objects, e.g., refrigerators in kitchens or computers in offices. There was also strong agreement between this categorization condition and a separate perception phase, where participants named the actual objects present in the natural scenes. For example, for an urban scene, the top three responses were the same whether participants were asked to name objects associated with urban scenes (categorization) or saw an image of an urban scene (perception): car, building, and people.

Furthermore, familiarity with a scene type appears to influence performance. The effect of familiarity was particularly evident in the continuous domain. Continuous category knowledge was measured via a task where participants estimated the average expected sizes, as well as minimum and maximum expected sizes, of real world objects (Hemmer and Steyvers, 2009a,b). Participants expressed stronger agreement for basic-level objects (e.g., apples) with which they were more familiar, and appeared to use knowledge about the common sizes of those objects to make size judgments. However, for less familiar objects, such as a chayote (a type of gourd), size estimates were closer to the superordinate category mean (e.g., fruits) such that participants appeared to use general knowledge at this category level to guide size judgments.

Categorical knowledge also extends to temporal events. People have strong expectations for the ordering of actions that form prototypical event sequences. Expectations for temporal events were measured by showing participants a sequence of events in achronological order and having them reconstruct the true order based on expectations, without seeing the true order of events (Hemmer et al., 2010). When asked to order well-defined temporal events, such as a wedding, they successfully reconstructed the chronological event sequence, despite the fact that the events were presented in no coherent order. This was contrasted with performance for events with undefined temporal order, such as random clay animations, where the ability to reconstruct the true events was no better than chance. This suggests that people are sensitive to the regularities of the ordering of actions within events.

Lastly, within the linguistic domain, knowledge for bi-directional categorical membership of color was measured via two tasks (Persaud and Hemmer, 2014): linguistic categorization (e.g., given a color swatch, name this color) and category representativeness (e.g., given a color name, generate what the color looks like). In the linguistic task, participants demonstrated hierarchical naming granularity for color categories, such that they expressed the strongest agreement for seven of the universal color categories – red, orange, yellow, green, blue, purple, and pink (Berlin and Kay, 1969; Uchikawa and Shinoda, 1996; Hardin, 2005; Xu et al., 2010; Webster and Kay, 2012) and a naming preference for 14 additional subordinate level labels within those categories (e.g., aqua-blue, light-blue, dark-blue). Importantly, the novel hue generating task showed a similarly strong agreement for hue values associated with the seven universal color categories, demonstrating a bi-directional relationship between the universal tendencies in color naming (Regier et al., 2005) and universality in color generation. This indicates that people have very detailed bi-directional knowledge of color that is highly reflective of the natural environment and that this category knowledge expresses a shared communicative value.

The findings from these studies clearly demonstrate that people are sensitive to the regularities of the environment and that this sensitivity influences performance in a variety of perceptual and cognitive tasks. Importantly, we argue that this influence also extends into the domain of episodic memory. In the next section, we explore how the influence of category knowledge contributes to episodic memory.

## WHAT IS THE ROLE OF CATEGORICAL KNOWLEDGE IN EPISODIC MEMORY?

Long-term episodic memory coding is known to rely on gist and meaning, including schemas (Bartlett, 1932; Rumelhart, 1980; Brewer and Treyens, 1981; Mandler, 1984), scripts (Schank and Abelson, 1977; Bower et al., 1979), and frames (Minsky, 1975). Over time, memory becomes more abstract and information is retained at this higher abstract level of knowledge (i.e., superordinate level). However, the cognitively preferred basic level of abstraction for categorization has also been shown to be a preferred level of retention in episodic memory (Pansky and Koriat, 2004). Thus, the relative contribution of category knowledge and how it operates on long-term episodic memory remains unclear.

A further dichotomy of the influence of category knowledge on episodic memory persists for reconstruction from memory. One standard finding in the literature is that categorical knowledge is a source of errors (e.g., Carmichael et al., 1932; Deese, 1959; Brewer and Treyens, 1981; Roediger and McDermott, 1995). For example, intrusions increased as a function of context, in the form of high prior expectations for objects consistent with natural scenes, e.g., books in offices, when those objects were not present (Brewer and Treyens, 1981). Similarly, studies of false memory, using the well-known Deese, Roediger, and McDermott paradigm (Roediger and McDermott, 1995), found intrusions of semantically associated but unstudied target words. However, such studies manipulate the naturalness of stimuli (e.g., the removal of books from offices), which in turn, impacts the usefulness of categorical knowledge. When experimental stimuli reflect the statistical regularities of the real world for which people’s knowledge is well-attuned, categorical knowledge can make a substantive contribution to episodic memory (see Steyvers and Hemmer, 2012 for a discussion on ecological validity).

Here we illustrate the relative contribution and the structure of influence of categorical knowledge on long-term episodic memory for naturalistic stimuli. An interesting finding was the relative contribution of categorical knowledge to reconstruction from memory (Hemmer and Steyvers, 2009c; Steyvers and Hemmer, 2012). While episodic information would be expected to make the greatest contribution to memory, guessing with categorical knowledge alone revealed a strong baseline contribution. In the discrete domain, participants recalled objects from natural scenes following a 2 or 10 s study interval. Accuracy in this task was quite high, with average accuracy above 85%, even after the sixth output position. Further, recall that the categorization task, outlined in the previous section, required participants to produce a list of objects associated with a given scene type. When these responses were evaluated against an actual image of that scene type (this can be thought of as a zero second study condition), average accuracy was above 70% for the first six responses. This difference between actual recall in the memory task and guessing with prior knowledge in the categorization task reveals the contribution of episodic memory. While it is not surprising that prior knowledge can be used to fill in the holes in noisy memory, it is unexpected that the baseline contribution of categorical knowledge alone would be so substantial.

The most striking finding of the effect of categorical knowledge is the average improvement in performance for stimuli associated with pre-experimental knowledge, relative to control stimuli not associated with pre-experimental knowledge (Hemmer and Steyvers, 2009a; Hemmer et al., 2010; Hemmer et al., in revision). Within the continuous domain, recall was better for familiar objects (i.e., fruits and vegetables), even when participants did not remember studying the objects, compared to unfamiliar objects (i.e., random shapes). Similarly in the temporal domain, 50% of participants achieved perfect or near perfect performance in the reconstruction from memory of the true order of events for which they had category knowledge, whereas only 5% achieved equivalent performance in the reconstruction from memory of events for which they had no prior expectations.

Furthermore, the contribution of categorical information to episodic memory reflects the granularity of category knowledge. A hierarchical influence is observed when knowledge is available at two or more category levels (Hemmer and Steyvers, 2009a; Persaud and Hemmer, 2014; Hemmer et al., in revision). In the continuous domain, recall for the sizes of objects was biased both by the mean size of the specific object studied (e.g., a pear) and by the mean size of all objects in the category (e.g., fruits). Smaller objects in the category (e.g., raspberries) were recalled to be larger and larger objects (e.g., pineapples) were recalled to be smaller. At the same time, an object studied at the small end of that object range (e.g., a small pear) was overestimated, whereas an object studied at the large end of that range (e.g., a large pear) was underestimated, independent of the absolute study size. This regression to the mean effect persists for recall of height as a function of gender (Hemmer et al., in revision), and recall of hue values associated with color categories (Persaud and Hemmer, 2014). Taken together, these findings show that categorical knowledge exerts a strong influence on reconstructive memory. In the subsequent section, we will discuss the optimality of such a strategy by the memory system.

## IS THIS AN OPTIMAL WAY FOR THE MEMORY SYSTEM TO WORK?

The reviewed studies suggest that the perceptual and memory systems systematically extract and exploit statistical regularities of the environment. Starting with his pioneering approach, Anderson (Anderson, 1989, 1990; Anderson and Milson, 1989; Anderson and Schooler, 1991) has argued that memory is optimized to make the best possible inference about the world given perceptual input. Episodic memory can be viewed as a problem of extracting and storing information from noisy signals presented to our senses, which need to be combined with knowledge about the structure of the environment. In the summarized studies, the use of stimuli for which people have categorical knowledge capitalizes on the idea that humans work in concert with their environment. People use knowledge to make sense of their environment, and appear to use this information optimally in a broad range of cognitive tasks.

A number of rational models have been developed in episodic and semantic memory (Anderson, 1990; Shiffrin and Steyvers, 1997; Steyvers et al., 2006; Steyvers and Griffiths, 2008; Xu and Griffiths, 2010) to characterize the computational problems people face when trying to make sense of the world given the sparse and noisy input from the senses. In categorical perception, Huttenlocher and colleagues presented a model of category effects in which immediate reconstruction from memory was based on a weighted average of episodic memory traces and prior knowledge in the form of category information (Huttenlocher et al., 1991, 2000).

The findings reviewed here have been well described by a rational model, assuming that reconstruction from memory uses multiple sources of information – episodic and categorical (Hemmer and Steyvers, 2009b; Persaud and Hemmer, 2014; Hemmer et al., in revision). Specifically, in these tasks, the goal of the observer is to reconstruct the original study events (sizes of fruits and vegetables, heights of people or hues of colors) as best as possible given noisy episodic memory content and prior knowledge of the stimulus attribute (i.e., size, height, or hue). This Bayesian approach gives a principled account of how knowledge of the world is combined with memory content to recall information about events. This model predicts systematic biases toward the category center, or prior category mean, at reconstruction, and a trade-off between the strength of knowledge and the strength of memory. When category knowledge is strong and memory representations are noisy, recall will more closely reflect category knowledge. Conversely, when knowledge is vague, but memory is strong, recall will more closely reflect the evidence stored in memory.

Similarly, when knowledge is available at two or more category levels, this trade-off corresponds to the intuition that for unfamiliar study features it is unlikely that category knowledge at the specific object level is reliable and inference instead reverts to a higher level categorical knowledge. Using prior knowledge at multiple levels of abstraction is an efficient strategy that allows generalization over experiences and correction of noisy memories. Assuming that noisy memory content is optimally combined with category knowledge of the environment, the memory system can exploit environmental regularities to “clean up” this noise and improve average recall performance.

## DISCUSSION

In this review, we have presented a perspective on the interrelatedness of categorical knowledge and memory. People have category knowledge and expectations that are closely aligned with the true environment. This knowledge plays a valuable role in memory retrieval and can increase the accuracy of memory. Furthermore, this interaction between episodic memory and structured knowledge representations is not an isolated phenomenon. These findings are robust and the interactions occur across multiple domains. While we have only reviewed findings in long-term episodic memory, there also appears to be an emerging understanding of the interrelatedness of category knowledge and visual short-term memory (Brady and Alvarez, 2011; Orhan and Jacobs, 2013), working memory and immediate memory (Heussen et al., 2011; Poirier et al., 2011). A potential area for furthering the understanding of the influence of categorical knowledge is the translational ability of categorical knowledge into novel domains. For example, a transfer of category knowledge has been demonstrated across visual and haptic modalities (Yildirim and Jacobs, 2013), and might be at the root of our ability to make predictions for novel domains.

Another area of interest is the developmental trajectory of the relationship between categorical knowledge and memory. Research in the developmental literature suggests that children, as young as 5 years old, employ categorical knowledge of the underlying stimulus distribution to improve recall performance (e.g., Duffy et al., 2006). What remains unclear is how children use categorical knowledge of naturalistic stimuli learned from the environment to aid recall, as demonstrated in adults, given their limited experience with the environment.

In contrast to assumptions of more traditional approaches in false memory research, that categorical knowledge leads to errors in recall, the sum total of the findings presented here suggests that this is not the complete story and instead categorical knowledge provides a beneficial source in episodic reconstruction. More broadly, convergence across realms of cognition spurs progress in the field, and advances our understanding of the cognitive system.

## AUTHOR CONTRIBUTIONS

Both authors participated in the initial and revised drafts of all sections of the manuscript. Both authors also contributed to the final draft of the manuscript and approved the manuscript for final submission.

## Conflict of Interest Statement

The authors declare that the research was conducted in the absence of any commercial or financial relationships that could be construed as a potential conflict of interest.

## Footnotes

1 We use the terminology categorical knowledge interchangeably with the concept of semantic memory/knowledge, but choose this term specifically because it is a subset of semantic knowledge, and the domain of knowledge that we are addressing in this review.
